# Editorial Department

**Published:** 1856-02

**Authors:** 


The "Virginia Medical and Surgical Journal" and the "Stethoscope," both published at Richmond, Va., have been combined under the name of the "Virginia Medical Journal." The first number of the new journal is very attractive, and in typographical appearance has no equal in the country.

The "Cincinnati Medical Observer" is the title of a new and handsome monthly, published by C. B. Stevens, M. D., and edited by professors George Mendenl.all and John A. Murphy. The first number promises well for its successors.

The most promising recent enterprize in medical journalism, is the "Louisville Medical and Surgical Review," which is to take the place of the "Western Journal of Medicine and Surgery." The first number will appear on the 1st of May next, under the editorial management of Prof. Gross and T. G. Richardson, M. D. How can it fail to to succeed? Dr. Gross has a surgical reputation wide enough and solid enough to make his journal sought for, while he has also the graceful, ready pen of a practiced writer. Talent, learning and industry combined, must make him one of the best editors in the country. His associate, Dr. Richardson, is the author of a work on Human Anatomy, said to possess great merit as a text book, though we have never seen it. We shall expect this new project to attain to a brilliant success.

